# 2-(1*H*-1,2,3-Benzotriazol-1-yl)-1-(4-ethyl­benzo­yl)ethyl 2,4-dichloro­benzoate

**DOI:** 10.1107/S1600536808011951

**Published:** 2008-04-30

**Authors:** Wu-Lan Zeng

**Affiliations:** a MicroScale Science Institute, Department of Chemistry and Chemical Engineering, Weifang University, Weifang 261061, People’s Republic of China

## Abstract

In the title mol­ecule, C_24_H_19_Cl_2_N_3_, the dihedral angles between the benzotriazole group and the ethyl- and dichloro-substituted benzene rings are 16.53 (1) and 82.09 (1)°, respectively. The crystal structure is stabilized by weak inter­molecular C—H⋯O inter­actions.

## Related literature

For related literature, see: Chen & Wu (2005 ▶). For bond-length data, see: Allen *et al.* (1987 ▶).
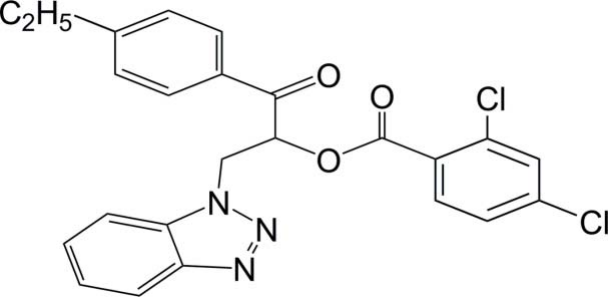


## Experimental

### 

#### Crystal data

C_24_H_19_Cl_2_N_3_O_3_
*M* *_r_* = 468.32
Triclinic, 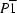

*a* = 9.251 (3) Å
*b* = 10.904 (4) Å
*c* = 11.057 (4) Å
α = 88.327 (6)°
β = 86.442 (6)°
γ = 83.304 (5)°
*V* = 1105.4 (6) Å^3^
*Z* = 2
Mo *K*α radiation
μ = 0.33 mm^−1^
*T* = 298 (2) K
0.20 × 0.20 × 0.10 mm

#### Data collection

Bruker SMART CCD area-detector diffractometer
Absorption correction: multi-scan (*SADABS*; Bruker, 1997 ▶) *T*_min_ = 0.938, *T*_max_ = 0.991
5375 measured reflections
3700 independent reflections
2701 reflections with *I* > 2σ(*I*)
*R*_int_ = 0.016

#### Refinement

*R*[*F* ^2^ > 2σ(*F* ^2^)] = 0.046
*wR*(*F* ^2^) = 0.124
*S* = 1.03
3700 reflections
289 parameters
H-atom parameters constrained
Δρ_max_ = 0.26 e Å^−3^
Δρ_min_ = −0.34 e Å^−3^

### 

Data collection: *SMART* (Bruker, 1997 ▶); cell refinement: *SAINT* (Bruker, 1997 ▶); data reduction: *SAINT*; program(s) used to solve structure: *SHELXS97* (Sheldrick, 2008 ▶); program(s) used to refine structure: *SHELXL97* (Sheldrick, 2008 ▶); molecular graphics: *PLATON* (Spek, 2003 ▶); software used to prepare material for publication: *SHELXTL* (Sheldrick, 2008 ▶).

## Supplementary Material

Crystal structure: contains datablocks global, I. DOI: 10.1107/S1600536808011951/lh2614sup1.cif

Structure factors: contains datablocks I. DOI: 10.1107/S1600536808011951/lh2614Isup2.hkl

Additional supplementary materials:  crystallographic information; 3D view; checkCIF report

## Figures and Tables

**Table 1:** Hydrogen-bond geometry (Å, °)

*D*—H⋯*A*	*D*—H	H⋯*A*	*D*⋯*A*	*D*—H⋯*A*
C11—H11*A*⋯O3^i^	0.93	2.51	3.425 (3)	169
C12—H12*A*⋯O1^ii^	0.93	2.52	3.378 (4)	154

Symmetry codes: (i) 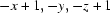; (ii) 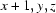.

**Fig. 1.**
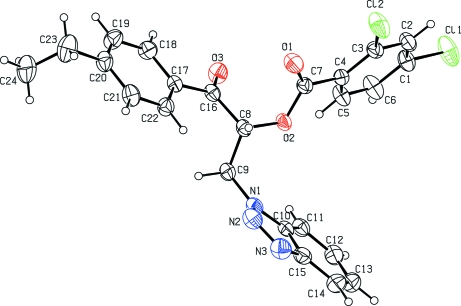
The molecular structure of (I), drawn with 30% probability ellipsoids.

**Fig. 2.**
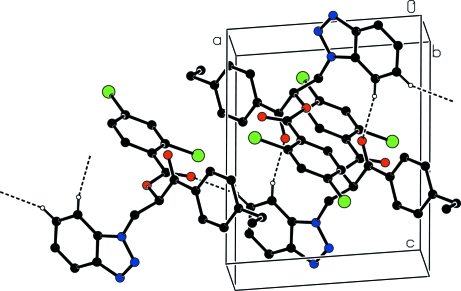
Part of the crystal structure of (I) showing hydrogen bonds as dashed lines. Only H atoms involved in hydrogen bonds are shown.

## supplementary crystallographic information

### Comment

1*H*-Benzotriazole and its derivatives are an important class of compounds because they exhibit a broad spectrum of pharmacological activities such as antifungal, antitumor and antineoplastic activities (Chen & Wu, 2005). All bond lengths and angles in the title molecule (I) are within normal ranges (Allen *et al.*, 1987). The benzotriazole ring system is essentially planar, with a dihedral angle of 1.05 (1)° between the triazole ring (atoms N1—N3/C10/C16) and the benzene ring (C10—C16). The dihedral angles between the mean planes of the benzotriazole system and ring atoms C1—C6 and C17—C22 are 82.09 (1) and 16.53 (1), respectively. The dihedral angle between rings atoms C1—C6 and C17—C22 is 89.47 (2). In the crystal structure, weak intermolecular C—H···O hydrogen bonds (Table 1) link molecules into chains extended along the *a* axis.

### Experimental

Bromine (3.2 g,0.02 mol) was added dropwise to a solution of 3-(1*H*-benzo[*d*][1,2,3]triazol-1-yl)-1-(4-ethylphenyl)propan-1-one (5.58 g,0.02 mol)and sodium acetate(1.6 g,0.02 mol) in acetic acid (50 ml). The reaction proceeded for 7 h. Water (50 ml) and chloroform (20 ml) were then added. The organic layer was washed successively with saturated sodium bicarbonate solution and brine, dried over anhydrous magnesium sulfate and the chloroform solution filtered. It was cooled with ice-water, and then an acetone solution (10 ml) of 2,4-dichlorobenzoic acid (3.8 g,0.02 mol) and tri ethylamine (2.8 ml) was added. The mixture was stirred with ice-water for about 6 h. The solution was then filtered and concentrated. Single crystals were obtained by slow evaporation of anacetone-ethylacetate(1:1 *v*/*v*) solution of (I) at room temperature over a period of one week.

### Refinement

All H atoms were located in difference Fourier maps and constrained to ride on their parent atoms, with C—H distances in the range 0.93–0.97 Å, and with *U*_iso_(H) = 1.2 *U*_eq_(C) and 1.5 *U*_eq_(methyl C) H atoms.

### Crystal data

**Table:**

C_24_H_19_Cl_2_N_3_O_3_	*Z* = 2
*M**_r_* = 468.32	*F*_000_ = 484
Triclinic, *P*1	*D*_x_ = 1.407 Mg m^−^^3^
Hall symbol: -P 1	Mo *K*α radiation λ = 0.71073 Å
*a* = 9.251 (3) Å	Cell parameters from 3700 reflections
*b* = 10.904 (4) Å	θ = 1.9–25.0º
*c* = 11.057 (4) Å	µ = 0.33 mm^−^^1^
α = 88.327 (6)º	*T* = 298 (2) K
β = 86.442 (6)º	Block, colorless
γ = 83.304 (5)º	0.20 × 0.20 × 0.10 mm
*V* = 1105.4 (6) Å^3^	

### Data collection

**Table:**

Bruker SMART CCD area-detector diffractometer	3700 independent reflections
Radiation source: fine-focus sealed tube	2701 reflections with *I* > 2σ(*I*)
Monochromator: graphite	*R*_int_ = 0.016
*T* = 298(2) K	θ_max_ = 25.0º
φ and ω scans	θ_min_ = 1.9º
Absorption correction: multi-scan(SADABS; Bruker, 1997)	*h* = −7→10
*T*_min_ = 0.938, *T*_max_ = 0.991	*k* = −12→12
5375 measured reflections	*l* = −10→12

### Refinement

**Table:**

Refinement on *F*^2^	Secondary atom site location: difference Fourier map
Least-squares matrix: full	Hydrogen site location: inferred from neighbouring sites
*R*[*F*^2^ > 2σ(*F*^2^)] = 0.046	H-atom parameters constrained
*wR*(*F*^2^) = 0.124	*w* = 1/[σ^2^(*F*_o_^2^) + (0.0528*P*)^2^ + 0.3617*P*] where *P* = (*F*_o_^2^ + 2*F*_c_^2^)/3
*S* = 1.03	(Δ/σ)_max_ < 0.001
3700 reflections	Δρ_max_ = 0.26 e Å^−^^3^
289 parameters	Δρ_min_ = −0.34 e Å^−^^3^
Primary atom site location: structure-invariant direct methods	Extinction correction: none

### Special details

**Table:**

Geometry. All e.s.d.'s (except the e.s.d. in the dihedral angle between two l.s. planes) are estimated using the full covariance matrix. The cell e.s.d.'s are taken into account individually in the estimation of e.s.d.'s in distances, angles and torsion angles; correlations between e.s.d.'s in cell parameters are only used when they are defined by crystal symmetry. An approximate (isotropic) treatment of cell e.s.d.'s is used for estimating e.s.d.'s involving l.s. planes.
Refinement. Refinement of *F*^2^ against ALL reflections. The weighted *R*-factor *wR* and goodness of fit *S* are based on *F*^2^, conventional *R*-factors *R* are based on *F*, with *F* set to zero for negative *F*^2^. The threshold expression of *F*^2^ > σ(*F*^2^) is used only for calculating *R*-factors(gt) *etc*. and is not relevant to the choice of reflections for refinement. *R*-factors based on *F*^2^ are statistically about twice as large as those based on *F*, and *R*- factors based on ALL data will be even larger.

### Fractional atomic coordinates and isotropic or equivalent isotropic displacement parameters (Å^2^)

**Table:**

	*x*	*y*	*z*	*U*_iso_*/*U*_eq_	
Cl1	0.63408 (12)	0.54323 (8)	0.16704 (8)	0.0986 (4)	
Cl2	0.17075 (9)	0.52373 (8)	0.46851 (10)	0.0910 (3)	
N1	0.5431 (2)	0.10230 (19)	0.83967 (17)	0.0471 (5)	
N2	0.4769 (3)	0.1341 (2)	0.9489 (2)	0.0609 (6)	
N3	0.5560 (3)	0.2042 (2)	1.0028 (2)	0.0672 (7)	
O1	0.1957 (2)	0.28033 (18)	0.58441 (18)	0.0651 (6)	
O2	0.41844 (18)	0.18987 (15)	0.61852 (15)	0.0468 (4)	
O3	0.3025 (2)	0.01135 (18)	0.51586 (16)	0.0608 (5)	
C1	0.5439 (3)	0.4634 (3)	0.2799 (2)	0.0596 (8)	
C2	0.4102 (3)	0.5146 (2)	0.3249 (2)	0.0577 (7)	
H2B	0.3685	0.5899	0.2942	0.069*	
C3	0.3380 (3)	0.4532 (2)	0.4161 (2)	0.0501 (6)	
C4	0.4002 (3)	0.3419 (2)	0.4646 (2)	0.0436 (6)	
C5	0.5355 (3)	0.2945 (2)	0.4167 (2)	0.0568 (7)	
H5A	0.5794	0.2204	0.4483	0.068*	
C6	0.6073 (4)	0.3530 (3)	0.3245 (3)	0.0694 (9)	
H6A	0.6977	0.3184	0.2924	0.083*	
C7	0.3240 (3)	0.2712 (2)	0.5603 (2)	0.0456 (6)	
C8	0.3506 (3)	0.1039 (2)	0.6972 (2)	0.0439 (6)	
H8A	0.2831	0.1483	0.7571	0.053*	
C9	0.4734 (3)	0.0290 (2)	0.7597 (2)	0.0482 (6)	
H9A	0.4352	−0.0383	0.8059	0.058*	
H9B	0.5452	−0.0065	0.6990	0.058*	
C10	0.6686 (3)	0.1556 (2)	0.8221 (2)	0.0431 (6)	
C11	0.7727 (3)	0.1540 (2)	0.7266 (2)	0.0503 (6)	
H11A	0.7664	0.1095	0.6570	0.060*	
C12	0.8851 (3)	0.2213 (3)	0.7409 (3)	0.0601 (8)	
H12A	0.9578	0.2228	0.6790	0.072*	
C13	0.8951 (3)	0.2882 (3)	0.8449 (3)	0.0700 (9)	
H13A	0.9738	0.3331	0.8504	0.084*	
C14	0.7928 (4)	0.2892 (3)	0.9380 (3)	0.0692 (8)	
H14A	0.7999	0.3340	1.0073	0.083*	
C15	0.6762 (3)	0.2204 (2)	0.9264 (2)	0.0538 (7)	
C16	0.2697 (3)	0.0221 (2)	0.6232 (2)	0.0431 (6)	
C17	0.1553 (3)	−0.0460 (2)	0.6839 (2)	0.0411 (6)	
C18	0.0778 (3)	−0.1156 (3)	0.6140 (2)	0.0562 (7)	
H18A	0.1012	−0.1206	0.5311	0.067*	
C19	−0.0327 (3)	−0.1769 (3)	0.6645 (3)	0.0643 (8)	
H19A	−0.0826	−0.2233	0.6154	0.077*	
C20	−0.0714 (3)	−0.1716 (3)	0.7863 (3)	0.0584 (7)	
C21	0.0066 (3)	−0.1038 (3)	0.8564 (3)	0.0606 (8)	
H21A	−0.0172	−0.0997	0.9392	0.073*	
C22	0.1188 (3)	−0.0418 (2)	0.8074 (2)	0.0514 (7)	
H22A	0.1699	0.0029	0.8572	0.062*	
C23	−0.1960 (4)	−0.2369 (3)	0.8398 (4)	0.0919 (12)	
H23A	−0.2398	−0.1926	0.9102	0.110*	
H23B	−0.2696	−0.2351	0.7808	0.110*	
C24	−0.1512 (5)	−0.3659 (4)	0.8759 (4)	0.1279 (18)	
H24A	−0.2344	−0.4024	0.9103	0.192*	
H24B	−0.0790	−0.3684	0.9349	0.192*	
H24C	−0.1110	−0.4112	0.8061	0.192*	

### Atomic displacement parameters (Å^2^)

**Table:**

	*U*^11^	*U*^22^	*U*^33^	*U*^12^	*U*^13^	*U*^23^
Cl1	0.1231 (8)	0.0765 (6)	0.0850 (6)	0.0018 (5)	0.0436 (6)	0.0298 (5)
Cl2	0.0503 (5)	0.0744 (5)	0.1377 (8)	0.0148 (4)	0.0167 (5)	0.0406 (5)
N1	0.0510 (13)	0.0529 (12)	0.0360 (11)	−0.0061 (10)	0.0035 (10)	0.0096 (9)
N2	0.0689 (16)	0.0705 (16)	0.0410 (13)	−0.0087 (13)	0.0113 (12)	0.0072 (11)
N3	0.0821 (19)	0.0766 (17)	0.0422 (13)	−0.0113 (15)	0.0051 (13)	0.0002 (12)
O1	0.0416 (12)	0.0720 (13)	0.0788 (14)	−0.0037 (10)	0.0036 (9)	0.0222 (10)
O2	0.0430 (10)	0.0462 (10)	0.0507 (10)	−0.0075 (8)	−0.0010 (8)	0.0142 (8)
O3	0.0685 (13)	0.0705 (13)	0.0441 (11)	−0.0188 (10)	0.0123 (9)	−0.0010 (9)
C1	0.074 (2)	0.0521 (16)	0.0493 (16)	−0.0051 (15)	0.0089 (14)	0.0100 (13)
C2	0.0620 (19)	0.0476 (16)	0.0618 (18)	−0.0022 (14)	−0.0054 (14)	0.0154 (13)
C3	0.0428 (15)	0.0477 (15)	0.0590 (16)	−0.0023 (12)	−0.0059 (12)	0.0075 (12)
C4	0.0450 (14)	0.0429 (14)	0.0434 (14)	−0.0080 (11)	−0.0032 (11)	0.0038 (11)
C5	0.0624 (18)	0.0464 (15)	0.0559 (17)	0.0070 (13)	0.0107 (13)	0.0119 (13)
C6	0.074 (2)	0.0603 (18)	0.0643 (19)	0.0143 (16)	0.0231 (16)	0.0125 (15)
C7	0.0416 (15)	0.0445 (14)	0.0501 (15)	−0.0042 (11)	−0.0027 (12)	0.0037 (12)
C8	0.0447 (15)	0.0441 (14)	0.0419 (13)	−0.0077 (11)	0.0042 (11)	0.0090 (11)
C9	0.0530 (16)	0.0463 (14)	0.0451 (14)	−0.0090 (12)	0.0010 (12)	0.0078 (11)
C10	0.0456 (15)	0.0429 (13)	0.0392 (13)	−0.0006 (11)	−0.0033 (11)	0.0097 (11)
C11	0.0472 (16)	0.0512 (15)	0.0501 (16)	−0.0002 (12)	0.0030 (12)	0.0073 (12)
C12	0.0459 (16)	0.0578 (17)	0.074 (2)	−0.0024 (14)	0.0039 (14)	0.0090 (15)
C13	0.0560 (19)	0.0633 (19)	0.092 (2)	−0.0130 (15)	−0.0131 (18)	0.0106 (18)
C14	0.083 (2)	0.0620 (18)	0.066 (2)	−0.0107 (17)	−0.0198 (18)	−0.0055 (15)
C15	0.0627 (18)	0.0527 (16)	0.0453 (15)	−0.0022 (14)	−0.0088 (13)	0.0043 (12)
C16	0.0414 (14)	0.0424 (13)	0.0429 (15)	0.0001 (11)	0.0055 (11)	0.0058 (11)
C17	0.0401 (14)	0.0385 (13)	0.0428 (14)	−0.0008 (11)	0.0028 (11)	0.0042 (11)
C18	0.0565 (17)	0.0672 (18)	0.0454 (15)	−0.0129 (14)	0.0013 (13)	0.0010 (13)
C19	0.0560 (18)	0.072 (2)	0.068 (2)	−0.0215 (15)	−0.0003 (15)	−0.0026 (15)
C20	0.0497 (17)	0.0519 (16)	0.073 (2)	−0.0095 (13)	0.0081 (14)	0.0057 (14)
C21	0.0667 (19)	0.0624 (18)	0.0506 (16)	−0.0116 (15)	0.0165 (14)	0.0087 (14)
C22	0.0572 (17)	0.0512 (15)	0.0466 (15)	−0.0130 (13)	0.0014 (12)	0.0018 (12)
C23	0.078 (2)	0.094 (3)	0.106 (3)	−0.039 (2)	0.028 (2)	0.000 (2)
C24	0.146 (4)	0.114 (3)	0.137 (4)	−0.077 (3)	−0.020 (3)	0.051 (3)

### Geometric parameters (Å, °)

**Table:**

Cl1—C1	1.729 (3)	C10—C11	1.384 (3)
Cl2—C3	1.720 (3)	C11—C12	1.360 (4)
N1—N2	1.356 (3)	C11—H11A	0.9300
N1—C10	1.358 (3)	C12—C13	1.393 (4)
N1—C9	1.438 (3)	C12—H12A	0.9300
N2—N3	1.298 (3)	C13—C14	1.354 (4)
N3—C15	1.379 (4)	C13—H13A	0.9300
O1—C7	1.194 (3)	C14—C15	1.399 (4)
O2—C7	1.349 (3)	C14—H14A	0.9300
O2—C8	1.432 (3)	C16—C17	1.479 (3)
O3—C16	1.213 (3)	C17—C18	1.384 (4)
C1—C6	1.368 (4)	C17—C22	1.388 (3)
C1—C2	1.366 (4)	C18—C19	1.368 (4)
C2—C3	1.376 (4)	C18—H18A	0.9300
C2—H2B	0.9300	C19—C20	1.372 (4)
C3—C4	1.389 (3)	C19—H19A	0.9300
C4—C5	1.378 (4)	C20—C21	1.376 (4)
C4—C7	1.482 (3)	C20—C23	1.506 (4)
C5—C6	1.365 (4)	C21—C22	1.378 (4)
C5—H5A	0.9300	C21—H21A	0.9300
C6—H6A	0.9300	C22—H22A	0.9300
C8—C9	1.511 (3)	C23—C24	1.471 (5)
C8—C16	1.517 (3)	C23—H23A	0.9700
C8—H8A	0.9800	C23—H23B	0.9700
C9—H9A	0.9700	C24—H24A	0.9600
C9—H9B	0.9700	C24—H24B	0.9600
C10—C15	1.379 (4)	C24—H24C	0.9600
			
?···?	?		
			
N2—N1—C10	109.9 (2)	C11—C12—C13	122.3 (3)
N2—N1—C9	119.9 (2)	C11—C12—H12A	118.9
C10—N1—C9	130.1 (2)	C13—C12—H12A	118.9
N3—N2—N1	109.1 (2)	C14—C13—C12	121.5 (3)
N2—N3—C15	107.8 (2)	C14—C13—H13A	119.2
C7—O2—C8	114.28 (19)	C12—C13—H13A	119.2
C6—C1—C2	121.2 (3)	C13—C14—C15	117.6 (3)
C6—C1—Cl1	120.3 (2)	C13—C14—H14A	121.2
C2—C1—Cl1	118.4 (2)	C15—C14—H14A	121.2
C1—C2—C3	119.2 (2)	N3—C15—C10	108.8 (2)
C1—C2—H2B	120.4	N3—C15—C14	131.5 (3)
C3—C2—H2B	120.4	C10—C15—C14	119.7 (3)
C2—C3—C4	121.0 (2)	O3—C16—C17	121.2 (2)
C2—C3—Cl2	116.5 (2)	O3—C16—C8	119.3 (2)
C4—C3—Cl2	122.5 (2)	C17—C16—C8	119.5 (2)
C5—C4—C3	117.6 (2)	C18—C17—C22	117.9 (2)
C5—C4—C7	119.7 (2)	C18—C17—C16	118.7 (2)
C3—C4—C7	122.7 (2)	C22—C17—C16	123.4 (2)
C6—C5—C4	122.1 (2)	C19—C18—C17	121.2 (3)
C6—C5—H5A	119.0	C19—C18—H18A	119.4
C4—C5—H5A	119.0	C17—C18—H18A	119.4
C5—C6—C1	118.9 (3)	C18—C19—C20	121.3 (3)
C5—C6—H6A	120.6	C18—C19—H19A	119.3
C1—C6—H6A	120.6	C20—C19—H19A	119.3
O1—C7—O2	122.2 (2)	C19—C20—C21	117.6 (3)
O1—C7—C4	126.3 (2)	C19—C20—C23	120.6 (3)
O2—C7—C4	111.5 (2)	C21—C20—C23	121.7 (3)
O2—C8—C9	105.67 (19)	C22—C21—C20	122.0 (3)
O2—C8—C16	109.74 (19)	C22—C21—H21A	119.0
C9—C8—C16	111.0 (2)	C20—C21—H21A	119.0
O2—C8—H8A	110.1	C21—C22—C17	119.9 (3)
C9—C8—H8A	110.1	C21—C22—H22A	120.0
C16—C8—H8A	110.1	C17—C22—H22A	120.0
N1—C9—C8	112.2 (2)	C24—C23—C20	113.1 (3)
N1—C9—H9A	109.2	C24—C23—H23A	109.0
C8—C9—H9A	109.2	C20—C23—H23A	109.0
N1—C9—H9B	109.2	C24—C23—H23B	109.0
C8—C9—H9B	109.2	C20—C23—H23B	109.0
H9A—C9—H9B	107.9	H23A—C23—H23B	107.8
N1—C10—C15	104.4 (2)	C23—C24—H24A	109.5
N1—C10—C11	132.5 (2)	C23—C24—H24B	109.5
C15—C10—C11	123.0 (3)	H24A—C24—H24B	109.5
C12—C11—C10	115.9 (3)	C23—C24—H24C	109.5
C12—C11—H11A	122.1	H24A—C24—H24C	109.5
C10—C11—H11A	122.1	H24B—C24—H24C	109.5

### Hydrogen-bond geometry (Å, °)

**Table:**

*D*—H···*A*	*D*—H	H···*A*	*D*···*A*	*D*—H···*A*
C11—H11A···O3^i^	0.93	2.51	3.425 (3)	169
C12—H12A···O1^ii^	0.93	2.52	3.378 (4)	154

Symmetry codes: (i) −*x*+1, −*y*, −*z*+1; (ii) *x*+1, *y*, *z*.
